# Self-insertion of needles: An unusual cause of empyema thoracis and its thoracoscopic management

**DOI:** 10.4103/0972-9941.59309

**Published:** 2009

**Authors:** Vinod Jain, Sandeep Tiwari, Samir Misra, Debashish Chaudhary

**Affiliations:** Department of Surgery, C.S.M. Medical University, Lucknow, India

**Keywords:** Empyema thoracic, intra-thoracic needles, video assisted thoracoscopic surgery

## Abstract

Intrapulmonary aberrant needles are rare in clinical practice. Most common cause till date is the intra-thoracic migration of pins and wires commonly used in treatment of fractures and dislocations of upper extremity. Some cases of traumatic intra-thoracic insertion of needles have also been reported. We report a patient of empyema thoracis due to unusual habit of self-insertion of needles in his body because of some myth. The patient was successfully managed by video-assisted thoracoscopic surgery.

## INTRODUCTION

Thoracic injuries due to intrapulmonary needles can result in acute complications like haemothorax, pneumothorax, pulmonary contusion, subcutaneous emphysema, thoracic wall laceration, pneumomediastinum and chronic complications like clotted haemothorax, chronic traumatic diaphragmatic hernia, aortic pseudoaneurysm, lung abscess, empyema, tracheo-oesophageal fistula.[1] Intrapulmonary aberrant needles are rare in clinical practice. Penetrating wounds in thoracic cavity often result in retention of metallic foreign bodies in the cavity.[1,2] Pins and wires commonly used in treatment of fractures or dislocations around the shoulder joint or clavicle may undergo intra-thoracic migration.[2,3]

We present a case with an unusual habit of self-insertion of needles, all over his body presenting as empyema.

## CASE REPORT

A 45-year-old male had a past history of 10 years of self-insertion of sewing needles all over his body, 1-2 needles per day for a period of 1½ years, in the myth that his neurologically ill daughter might get cured by this manouever. During this period of 10 years, he was asymptomatic except the two episodes of abscess formation, one in left back about 5 years back and another in left forearm 3 years back.

**Figure 1:**
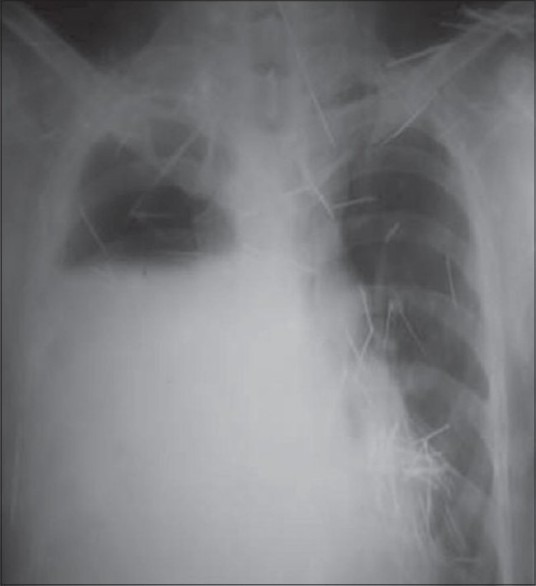
Preoperative chest X-ray

He presented to our institute with a history of swinging pyrexia, cough with expectoration, right-sided chest pain and with ICD *in situ* along with broncho-pleural fistula (BPF). Clinical evaluation revealed anaemia, malnurition and dyspnoea and diminished air entry in the right lung. Routine blood reports were in normal range except Hb 4.5 g%, TLC 24,500/mm^3^, DLC P_90_L_10_E_0_M_0_. Pus culture revealed *Staphylococcus aureus*, sputum for AFB and serum IgA, IgG IgM for TB negative. An x-ray of the chest [Figure 1] showed right pyopneomothorax, a collpased lung, an intercostal drainage tube in place and multiple metallic foreign bodies. X-rays of upper and lower extremities also showed multiple metallic foreign bodies. Psychiatric evaluation was done at our institute and found nothing abnormal. The patient was never evaluated for any psychiatric illness before, neither he was taking any medication for any such illness.

During initial management, the ICD was repositioned, toxemia controlled and nutrition supplemented. During this period of 1 month, BPF healed and then the patient was taken up for video-assisted thoracoscopic surgery (VATS).

**Figure 2:**
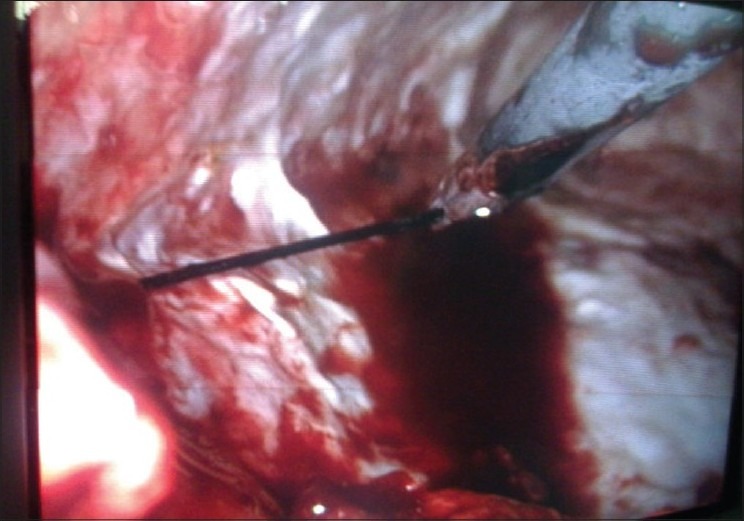
Intraoperative image

**Figure 3:**
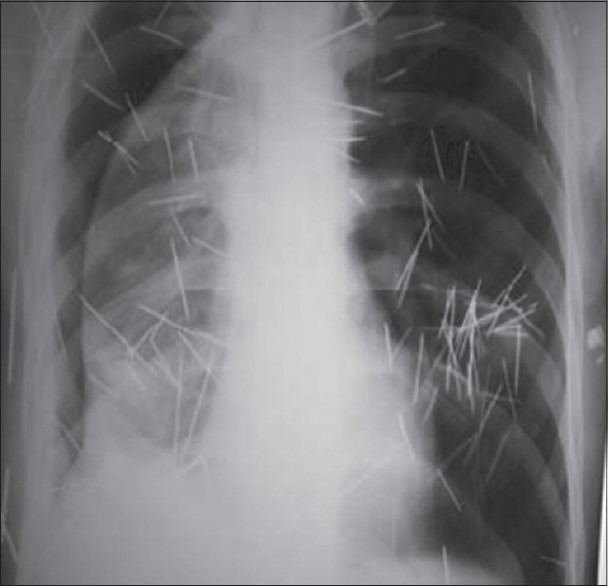
Postoperative chest X-ray after 10 days

The patient was operated under single lung general anaesthesia using double lumen endotracheal tube in a typical right thoracotomy position. Three-port thoracoscopy was done and telescope was inserted through the ICD site. Intraoperative findings revealed synechiae and pus collection. The synechiae were broken, pus drained and thoracic cavity lavaged with normal saline. Six needles were found and all were removed from pleural cavity [Figure 2] (two freely floating in pleural cavity, three projecting through lateral chest wall and one embedded in visceral pleura). After this, ICD was again placed in thoracic cavity. The patient was discharged on third post-operative day, and his ICD was taken out after 10 days. Post-operative X-ray (PA and lateral views) after 10 days [Figure 3] and follow-up after 3 months clearly showed near normal lung expansion and the metallic needles which were embedded in chest wall only. His follow-up PFT also improved with FEV_1_-68% and FVC-2.5 l.

## DISCUSSION

Intra-thoracic migration of pins used in orthopaedic fixation is a well-described phenomenon,[2,3] but self-insertion of needle is very unusual. Various explanations have been suggested for this migration, which include muscular activity, respiratory exertion, capillary action, gravitational forces and negative intra-thoracic pressures.[2,3] Various catastrophic complications of migrated pins have been reported like pericardial temponade, arrhythmias, pericarditis, false aneurysm, aorto- pulmonary fistulae, pneumothorax and haemoptysis.[3,4] These are similar to that of penetrating chest trauma.[1] Chronic complications include clotted haemothorax, chronic traumatic diaphragmatic hernia, aortic pseudoaneurysm, empyema[1,5] and bronchoesophageal fistula.[1]

In patients with penetrating chest trauma, foreign bodies and its complications can be detected by chest radiographs, USG and CT scan. These are also used for post-operative follow up in such cases.[5,6]

This patient, as he was asymptomatic for 10 years except for his two abscesses, he never consulted a doctor. He consulted only when he developed empyema, which is a well-known complication of retained intra-thoracic foreign body.[1,5] After being treated elsewhere for past 1½ months, he came to our institute with ICD *in situ*. Initially on admission, his preoperative evaluation was done. Routine investigations for tuberculosis were negative, chest radiographs (PA view) showed pyo-pneumothorax on right side with metallic foreign bodies scattered all over the chest and other parts of the body. PFT showed restrictive pattern. The patient was initially managed conservatively and when improved, VATS was performed.

VATS is now a well-established technique in the armamentarium of the thoracic surgeons. Jacobacus is credited as the first person for clinical application of thoracoscopy. He performed adhesiolysis to enhance pneumothorax therapy of tuberculosis via a cystoscope introduced into the pleural cavity.

In this case, the patient was operated by VATS with single lung ventilation using double lumen endotracheal tube. Under thoracoscopic guidance, pus was drained, synchiae broken, foreign bodies retrieved and the patient was put on chest physiotherapy. The patient improved, as showed by his improved general condition, post-operative chest radiographs and PFTs.
